# A feasibility study and pilot randomised trial of a tailored prevention program to reduce falls in older people with mild dementia

**DOI:** 10.1186/1471-2318-13-89

**Published:** 2013-09-03

**Authors:** Jacqueline Wesson, Lindy Clemson, Henry Brodaty, Stephen Lord, Morag Taylor, Laura Gitlin, Jacqueline Close

**Affiliations:** 1 Ageing Work and Health Research Unit, University of Sydney, Lidcombe, Australia; 2 Dementia Collaborative Research Centre, Better Assessment, Randwick, Australia; 3 Centre for Healthy Brain Ageing, School of Psychiatry, University of New South Wales, Randwick, Australia; 4 Neurosciences Research Australia, University of New South Wales, Randwick, Australia; 5 Johns Hopkins University, Baltimore, USA; 6 Prince of Wales Clinical School, University of New South Wales, Randwick, Australia; 7 Centre for Population Ageing Research (CEPAR), Sydney, Australia

**Keywords:** Accidental falls, Dementia, Aged, Community based, Feasibility, Occupational therapy, Physiotherapy

## Abstract

**Background:**

People with dementia have a disproportionately high rate of falls and fractures and poorer outcomes, yet there is currently no evidence to guide falls prevention in this population.

**Methods:**

A randomised trial design was used to test feasibility of study components and acceptability of a home hazard reduction and balance and strength exercise fall prevention program. The program was tailored to participant’s individual cognitive levels and implemented as a carer-supported intervention. Feasibility of recruitment, retention and implementation of intervention were recorded through observation and documented in field notes. Adherence, carer burden and use of task simplification strategies were also monitored. Outcome measures, collected at 12 weeks included physiological, fear of falling, cognitive and functional measures.

**Results:**

Recruitment was achievable but may be more challenging in a multisite trial. Twenty two dyads of persons with mild dementia and their carers were randomised to intervention or usual care control group. Of 38 dyads referred to the study, there was a high rate of willingness to participate, with 6 (16%) declining and 10 (26%) not meeting inclusion criteria. The intervention was well received by participants and carers and adherence to both program components was very good. All participants implemented some home safety recommendations (range 19-80%) with half implementing 50% or more. At the end of 12 weeks, 72% of the intervention group were exercising. Both the rate of falling and the risk of a fall were lower in the intervention group but these findings were not significant (RR= 0.50 (95% CI 0.11-2.19). There were no differences in physiological outcome measures between the control and intervention groups. However results were influenced by the small study size and incomplete data primarily in the intervention group at follow up.

**Conclusions:**

The pilot study was feasible and acceptable to people with mild dementia and their carers. The lessons learnt included: recruitment for a larger trial will require multiple approaches; home safety recommendations should provide a greater emphasis on environmental use compared with behavioural change; strategies to ensure an adequate dosage of exercise should be further explored. We recommend that intervention delivery incorporate an integrated occupational therapy and physiotherapy approach and that carers be provided with an individualised session to enhance dementia-specific skills in management and communication. A refined intervention should be tested in a randomised trial with an adequately powered sample size.

**Trial registration:**

Australia and New Zealand Clinical Trials Registry 126100001049066

## Background

Dementia is a major health care problem and affects approximately 6-7% of community-dwelling older people [1,2]. The medical and social costs of dementia, estimated as 1.4% of GDP, is projected to increase by 85% by 2030 with approximately 60% of people with dementia living in the community [1,2]. Dementia has been consistently shown to be associated with an increased risk of falls, with rates double that of cognitively intact older people [3,4]. A fall related event with or without fracture is the most common reason for hospitalisation in people with dementia, accounting for approximately 26% of all admissions [5].

There is robust evidence to support interventions for preventing falls in older people [6] involving both strength and balance training and home hazard reduction. A meta-analysis of exercise studies showed a 17% fall reduction (pooled rate ratio = 0.83 CI 0.75 to 0.91) with the greatest effect (42% fall reduction) from programs that provide high level balance challenge and in sufficiently high dosages of prescribed exercise [7]. A strong effect (39% fall reduction) for home safety interventions has also been demonstrated in a meta-analysis with at-risk persons (pooled rate ratio = 0.61 (0.47 to 0.79) [8].

However, there is currently no evidence to guide fall prevention in older community dwelling people with dementia, despite these people having a disproportionately high rate of falls and fractures and poorer outcomes [9]. One trial that specifically targeted people with dementia using multi-factorial assessment and referral to community services was not successful in preventing falls [10], and another trial of a tailored multi-factorial intervention which was not effective overall, reduced falls in a sub-set of 70 people with an MMSE of < 27 who lived with a carer [11]. Physical activity interventions have been tested in people with mild to moderate cognitive impairment but these did not specifically target fall prevention [12,13].

Our aim was to conduct a pilot randomised controlled trial exploring the design and feasibility of a novel approach to fall prevention for people with mild dementia living in the community. We tested the feasibility of the study components and the acceptability of a home safety and exercise fall prevention program tailored to individual cognitive levels and implemented as a carer-supported intervention.

## Methods

### Study design

We used a 12 week randomised pilot trial with single-blinded outcome assessment to explore the feasibility of recruitment, study procedures, outcome measures, retention and the implementation of a novel intervention. The unique approach involved tailoring the delivery of the intervention according to participants’ preserved cognitive abilities as measured by Allen’s Cognitive Disability Model [14]. The intent was to inform the protocol to be used in a future larger randomised trial. The study protocol was approved by the South Eastern Sydney Illawarra Area Health Service Human Research Ethics Committee (HREC Reference Number 09/063).

### Setting and participants

A convenience sample of dyads of people with mild dementia and their carers was recruited from a Memory Disorders, a Cognitive Disorders and an Aged Care Clinic, and a clinical dementia service network within the local health network in the eastern suburbs of Sydney, Australia. Eligible participants were community dwelling people over 65 years of age with a specialist diagnosis of dementia or an Addenbrooke’s Cognitive Examination (ACE-R) score [15] of ≤82 indicating the cut off score for dementia. The ACE-R was administered at baseline. Participants also had a non-paid carer (usually a family member) with a minimum of 3.5 hours per week of face to face contact. Carer participation was essential for the delivery of the intervention and for assisting with the recall of falls. Participants had to be English speaking given that all assessments and interventions were conducted in English. Exclusion criteria included delirium or an acute medical condition; severe psychiatric disorder or progressive neurological disorder (except dementia); an MMSE < 12 (given the likely difficulties of following simple commands); severe visual impairment (as visual cues were utilised to enhance uptake of exercises); and residents of aged care facilities. Informed consent was obtained from each participant and their carer.

### Randomisation and assessor blinding

Randomisation was conducted by an investigator not involved in assessment or intervention, using a random numbers table and permuted blocks of four and six. Group allocation was concealed using opaque, sealed envelopes with study identification number in sequential order. Baseline assessments were completed by the occupational therapy interventionist together with a research assistant for physical measures to ensure participant safety during assessment. Participants were then allocated to the intervention group or the usual care control group. Assessors blinded to group allocation were used to complete follow up assessment at four months.

### The intervention

**Figure 1:**
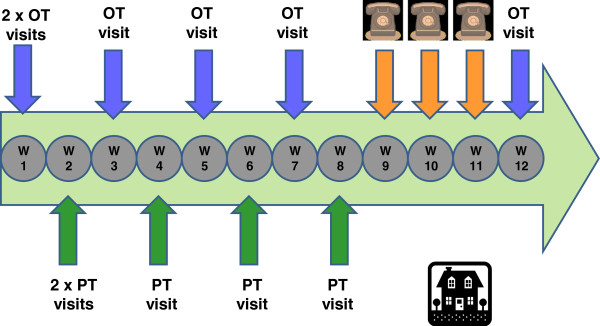
Intervention schedule: occupational therapy (OT), physiotherapy (PT) visits and phone calls over 12 weeks.

The intervention consisted of strength and balance training exercises and home hazard reduction. The 12 week intervention program was conducted in participants’ homes. Figure 1 outlines the intervention schedule of occupational therapy (OT), physiotherapy (PT) home visits and three telephone calls over 12 weeks. The occupational therapist (OT) was project manager, monitored overall program adherence, conducted functional cognition assessments, completed home safety assessments and prescribed home safety recommendations, and helped carers implement home safety recommendations. The physiotherapist prescribed and progressed the exercises, and monitored adherence. Carers were considered partners in care: they supervised exercise sessions and were generally responsible for implementation of home safety recommendations. The OT also discussed behaviour and/or management issues with carers and strategies were provided such as task simplification, modifying the environment, and education about participants’ cognitive abilities.

### Functional cognition

The intervention used Allen’s Cognitive Disabilities Model [14] to tailor the adaptation and delivery of the exercises and home safety fall prevention interventions. Participants were assessed by the occupational therapist in week 1 using the Large Allen’s Cognitive Levels Screening Tool-5 (LACLS-5) [16]. This measure is designed to identify global levels of functional cognition and participants’ capacity to perform routine tasks and adapt to novel situations. The tool objectively evaluates cognitive level according to participants’ abilities on a novel leather lacing task which is increasingly complex with graded instructions provided by the assessor according to abilities observed. The LACLS-5 takes from 15–30 minutes to administer and validation with an additional task can take a similar period of time. While six cognitive levels are defined within Allen’s model, each with specific assets and limitations (26 discrete levels or performance modes), levels 3.0 to 5.8 are assessed using the LACLS-5 tool. The LACLS-5 scores are hypothesised to reflect the problem solving abilities described by the levels and performance modes. A quick estimate of functional cognition is provided, and an earlier version of the screen has been found to have high predictive value in determining capacity for functional living skills and the need for assistance across living environments [17].

### Home safety intervention

The Westmead Home Safety Assessment [18] was used as a tool by the occupational therapist, the carer and the person with dementia, to audit the home environment systematically for environmental and behavioural fall hazards. Participants were provided with a booklet of home safety recommendations which formed the basis of subsequent OT visits. Recommendations were tailored to the specific hazards identified in participants’ homes and the format was adapted to cognitive abilities. The booklet included a description of the selected hazards, explanation of why situations were hazardous, and sections with habits to change, items to buy and, if required, home modification service referrals. Some smaller items, such as sensor lights were provided by the OT investigator and trialled before participants purchased them.

The design of the home safety booklet was modified according to Allen’s theory [14]. For example, the reasoning around the hazard identification was explicitly stated for each recommendation because people functioning at level mode 4.4 do not anticipate hazards or secondary effects of actions, and they are not likely to identify implied relationships, for example, that dizziness may be a symptom of other causes, not only a result of standing too quickly. The section detailing habits to change made explicit the behaviours that carers may need to train: people at level 4 are expected to be able to learn new routines with 2–3 weeks of supportive, consistent practice. People below level mode 4.6 do not scan the environment effectively and may demonstrate ‘tunnel vision’ when moving about their environment so recommendations commonly included fluorescent step edges, removal of below knee height hazards and re-organising furniture to allow improved access. While some recommendations, such as improved lighting, are commonly prescribed, knowledge of cognitive level allowed tailored prioritisation and additional reasoning to be discussed with carers.

### Exercise intervention

Each participant was prescribed up to six individually tailored strength and balance exercises which were selected from the Weight-Bearing Exercise for Better Balance (WEBB) program [19] and based on the results of the physical performance assessment. The intervention commenced after information had been gathered in relation to physical and cognitive function so as to ensure that the approach used to deliver the exercises was consistent with the physical and cognitive abilities of the participant. For example, participants functioning at level 3 needed full carer supervision and demonstration of every action repeated in each set of exercises, so as to copy the action, as they were unable to follow written instructions at this level. Participants at level 4 have strong interest in visual cues in the environment and this was incorporated into the intervention by identifying visual cues in the home to use while exercising and ensuring the written booklet was adapted to capture participants’ attention. Participants at level 5 were able to take on new learning with instruction and were able to complete aspects of the program more independently and exercise without supervision of carers.

The strength training exercises included sit to stand, calf raises and step ups onto a block. Static balance tasks included a series of stance positions with diminishing base of support (i.e. standing with feet together, semi tandem, near tandem and tandem) with eyes open or closed. Dynamic balance exercises included stepping over a strip of matting on the floor, foot taps onto a block, lateral side steps, sideways walking and step ups. Possible progression included increased frequency, increased repetitions, decreased chair height, increased time held in balance stances and advancement to more difficult static balance tasks, reduced support (removing UL support) or increased height of stepping block.

A booklet was provided containing the prescribed strength and balance exercises. Modifications to the format included large print and colour contrasted fonts to enhance appearance and highlight important information, colour photographs (not stick figures or sketches) of correct technique and simplified written instructions (including where to complete exercises, number of repetitions). Exercises were always completed in the same place within participants’ homes and details, for example of where to stand or hold on, was recorded on each exercise sheet. Adherence and adverse events were also recorded in this booklet. When exercises were progressed, new exercise sheets were inserted and old ones removed so as to retain the simple structure.

### Control group

Participants in the control group received ‘usual care.’ They were encouraged to report any falls to their general practitioner and did not receive any further contact from the investigators except for collection of falls data and follow up assessment. Both intervention and control groups received health promotion brochures on fall prevention and home safety.

### Study measures

#### Recruitment, retention and acceptability of study components and intervention

Referral source and reasons for exclusion or declining were documented. Adherence to the intervention protocol was recorded using field notes during each visit and included comments regarding acceptability of study components – whether participants were engaged in the exercise and/or home safety interventions. Exercises were monitored using a checklist in the exercise booklet completed by participants (if exercising unsupervised) or carers and percentage of home safety recommendations implemented with dates recorded by the OT in the home safety booklet.

### Clinical outcome measures

Assessments took place at baseline prior to randomisation and at follow-up four months later. Carers were present at baseline and follow-up assessments which included demographics, medical history and fall history for prior 12 months.

### Everyday ability measures

The following measures were completed through interviews with carers: daily functioning using the Interview for Deterioration of Daily Activities in Dementia (IDDD) [20], mood using the Cornell Scale for Depression in Dementia [21], and behaviour using the Agitated Behaviours in Dementia Scale [22]. Physical activity levels of participants were measured using the Incidental and Planned Exercise Questionnaire – weekly (IPEQ-W) for older people [23] where total amount of hours spent doing planned and incidental physical activities, including self care, home maintenance and exercise were recorded.

### Carer measures

Carer burden was measured with the Zarit Burden Interview (short form) [24,25] and carers’ ability to simplify everyday self care tasks for people with dementia was measured using the Task Management Strategy Index (TMSI) [26].

### Physical performance

The Physiological Profile Assessment (PPA) [27] was used to assess physiological performance. The PPA contains five validated measures of physiological function for people with intact cognition: visual contrast sensitivity, proprioception, quadriceps strength, simple reaction time, and postural sway while standing on a foam rubber mat with eyes open. The resulting fall risk score can predict the risk of recurrent falls with 75% accuracy over a 12-month period. Other physical measures included the Hill Step Test [28] and the near-tandem test of standing balance with eyes closed [13]. The Hill Step Test provides a measure of dynamic balance. Dynamic balance is not measured by the PPA or the near tandem eyes closed balance test, dynamic balance involves shifting weight to enable everyday activities and walking. Step tests have also been associated with fall risk [29]. Performance on these physical assessments also provided the basis for exercise prescription.

### Falls

Fear of falling was assessed with the Falls Efficacy Scale - International (Short Form) [30] and the Iconographical Falls Efficacy Scale – International (ICONFES) [31]. Falls, defined as unintentionally coming to rest on a lower level, were monitored using monthly falls calendars completed by the carer. If calendars were not returned or if a fall was reported, an investigator telephoned the carer to obtain details.

### Statistical analysis of falls and clinical measures

Data were analysed using SPSS 19.0 for Windows (SPSS, Inc., Chicago, IL). Frequencies and histograms provided exploration of individual changes. Data were analysed using intention to treat and without imputation for missing data because of the exploratory nature of the pilot. Differences between groups for rate of falls were compared with Incident Rate Ratios using the negative binomial regression model and for number of fallers using a relative risk (RR). For other measures, change scores were generated. Due to the small sample size and because the data were skewed, outcome trends were analysed using the Mann–Whitney *U*-test.

## Results

### Recruitment and sample characteristics

Recruitment commenced by the RA/interventionist in June 2010 and continued for six months.

**Figure 2:**
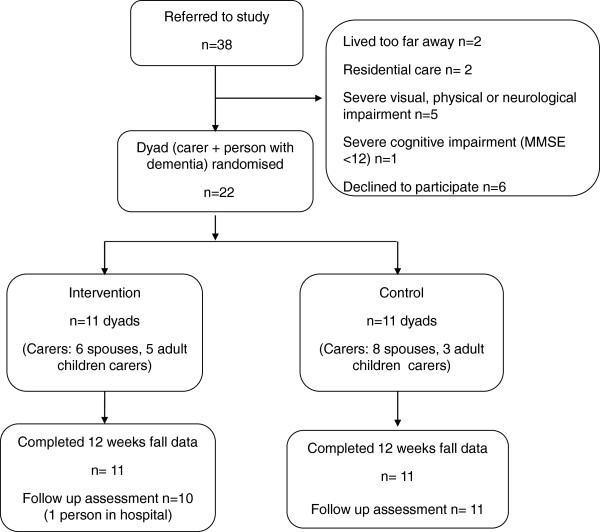
Flow chart of participants through the trial.

Of the 38 dyads referred to the study, there was a high rate of willingness to participate, with only 6 (16%) declining and 10 (26%) not meeting inclusion criteria. Reasons for exclusion were: living too far from study centre (n=2), living in residential care (n=2), having severe vision impairment (n=3), a degenerative neurological condition (multiple sclerosis) (n=1), severe pain/physical disability (n=1), or severe cognitive impairment (n=1). Six people declined participation. Reasons included not interested (n=2), lack of carer time (n=2), carer view that intervention was not necessary (n=1) and timing did not suit the carer (n=1). Twenty two (58%) dyads of carer and participant were randomised into the intervention (n= 11) or control groups (n= 11) and followed up for four months. The flow of participants through the trial is shown in Figure 2.

There were no significant differences at baseline between the intervention and control groups in terms of demographic characteristics or assessment measures (Table 1). We examined cognitive function in the 13 (59%) carers who were 65 years or over: mean MMSE was 28.7 (SD=1.9), with a range of 24–30.

**Table 1:** Baseline characteristics of participants with dementia

**Baseline characteristics**	**Intervention (n=11)**	**Control (n=11)**
Gender: n (% women)	5 (45.5%)	4 (36.4%)
Age (mean ±SD)	78.7 (± 4.2)	80.9 (± 5.0)
Years of education (mean ±SD)	10.6 (±2.4)	12.0 (±4.3)
Number of medications (mean ±SD)	3.4 (±1.7)	5.0 (±2.5)
Number of co-morbidities (mean ±SD)	3.0 (±2.4)	4.1 (±2.2)
Living situation:		
Lives alone^a^ n (%)	4 (36.4%)	2 (18.2%)
Lives with spouse or family n (%)	7 (63.6%)	9 (81.8%)
Female carers (%)	8 (72.7%)	8 (72.7%)
Falls previous 12 months (mean ±SD)	2.09 (±2.5)	2.45 (±3.2)
Number falls in past year:		
No falls	4 (36.4%)	2 (18.2%)
1 fall	2 (18.2%)	4 (36.4%)
2 or more falls	5 (45.5%)	5 (45.5%)
ACE-R^b^ (mean ±SD)	67.8 (±12.6)	62.5 (±14.2)
MMSE^c^ (mean ±SD)	24.5 (±3.1)	22.5 (±4.3)

Notes:

^a^Carers were adult children of participants who met inclusion criteria of at least 3.5 hours face to face contact per week.

^b^ACE-R – Addenbrooke’s cognitive examination – revised. Score range 0–100. Cut off score for dementia ≥82.

^c^MMSE – Mini mental state examination. Score range 0–30.

Higher scores on both ACE-R and MMSE indicate better cognition.

All dyads completed all measures at baseline assessment. All dyads in the control group had a follow-up assessment, with the majority of measures completed. One dyad in the intervention group was not available for follow-up as the participant was in hospital with a leg ulcer. The remaining 10 dyads (91%) were available but not all measures were completed: one person completed only the physical measures, one person declined to complete more than three measures for unspecified reasons and one person’s carer did not return measures.

The intervention group received a mean number of 10 home visits (range 6–12) of 57.5 minutes duration and 3.5 telephone calls of 12.9 minutes duration, equating to 10.3 hours of direct contact per participant. Two people (18%) had fewer than ten visits: one person had only 6 visits as she declined the home safety intervention and the other was unwell after the 7^th^ visit and discontinued intervention.

### Cognitive status

All participants demonstrated cognitive impairment in the mild range, as shown by the MMSE mean scores (Table 1) and ranges (intervention 20–29; control 15–28). There was no statistical difference between groups (t (20) = .92, p>.05). Functional cognition in the intervention group, as assessed with the LACLS-5 was within the expected range for people with dementia living in the community with mean and median scores for the intervention group being 4.4 (range 4.2 – 5.6). At the 4.4 level of function behaviour is goal directed but people do not visually scan their environment, do not anticipate hazards and cannot solve problems independently, so it is not recommended that they live alone [14]. The four participants below this level lived with spouses.

### Adherence to recommendations for home hazard reduction

One dyad withdrew from the home safety intervention as they were not interested in having their home assessed. There was a mean of 21.2 (range 13–31) hazard reduction recommendations and the remaining 10 participants implemented some changes to reduce hazards (range 19–80%). Fifty percent of participants (n=5) implemented 50% or more of the recommendations.

The most commonly adopted recommendations were the simple changes, such as changing footwear and rearranging small items of furniture, and recommendations completed by the therapist during visits, such as double sided tape securing mats, fluorescent tape to highlight step edges and using sensor lights. Recommendations for behaviour change related to habitual behaviours, such as not leaving bags on floors to trip over straps. Older participants (>80 years) (and carers) who had multiple falls prior to the study (n=3, 30%) were the most enthusiastic about changes and had the highest rate of implementation. Overall, there were trends to higher adherence (implementing 70-80%) for those with a history of falls and a poorer cognition (lower ACE-R scores). Higher carer stress tended to result in lower adherence.

### Adherence to exercise intervention

All participants in the intervention group (n=11) were prescribed between 2 and 6 exercises (mean 4.3) at the start of the program, and these were to be completed three times per week. Recommendations regarding frequency of exercise sessions and repetitions for each exercise were lowest for one participant who had been inactive for a long period of time prior to study commencement (n=3 exercises) and for another who lived alone and was not able to exercise without supervision (n= 2 exercises). Initial exercise prescription erred on the side of low intensity for all participants which may have been less challenging for those more physically capable, for example, one participant who routinely attended a gym 3–4 × week.

The number of exercises prescribed increased to a mean of 5.5 (range 4–6) by 12 weeks. All participants were prescribed: at least one static balance exercise, with the majority (n=7, 63%) prescribed two balance exercises (range 1–2). Nine participants (82%) were prescribed one or more dynamic balance exercises (range 0–3). All participants were prescribed between one and three strength exercises with four participants (36%) prescribed three strength exercises. Participants were progressed between 2–4 occasions (mean 3) but not every exercise was upgraded on each occasion. All participants were progressed by increasing the number of repetitions for selected exercises (increased by 2, 5 or 10 repetitions) and/or increasing level of difficulty of exercise (for example narrowing base of support in semi tandem to near tandem then adding tandem stand (eyes open) or increasing the time in the exercise stance (range 5 to 30 seconds) by 5–15 seconds). No participants were prescribed weights but used gravity for resistance such as in sit to stand, and three (22.3%) were prescribed step-ups with increasing step height.

Carers provided supervision of the exercises in 63.6% (n=7) of cases. One participant did not require supervision as he functioned at cognitive level mode 5.2 and was deemed to be able to complete exercises independently. Two male participants did not want their spouses to supervise and one spouse-carer considered that the participant did not need supervision. These participants demonstrated correct technique and were supported by the individualised cueing and instructive exercise booklets.

According to adherence records in the exercise booklets and verification during visits by study staff, participants completed exercises a mean of 2.8 times per week with a range of 0 to7 (SD ± 1.4). Brief physical illnesses, such as gastroenteritis and mild joint pain (n=5) and holidays (n=3) reduced adherence for periods of up to two weeks during the intervention time frame for eight (72.7%) participants. At the end of the intervention period 72.7% (n=8) participants were still completing exercises (mean = 3.3, range 2–5 times per week), with prolonged ill health, hospitalisation and/or residential care placement the reasons for stopping (n=3). Availability of carers to supervise exercise limited the exercise prescription in three cases (27%) with only twice weekly sessions being possible when they did not live with the participant. All carers and participants reported enjoyment and satisfaction with the program. One participant and a number of carers (n=4; 36%) commented that they believed the exercises could have been stronger/ more intense than those prescribed.

### Safety and adverse events

No serious adverse events related to the intervention were reported during the study period. Minor complaints relating to stiffness, dizziness and mild joint pain (n=4; 36%) were reported by participants intermittently and exercises were adjusted accordingly.

### Clinical outcome measures

Interquartile ranges demonstrate high variability in both groups in this small sample. There was differential loss of data at follow up with more loss occurring in the intervention group (see Table 2). Five people (23%) did not complete the IDDD or ICON-FES scales at follow up and 7 (32%) did not complete the Hill Step Test. There were no significant differences between groups for any of the outcome measures at retest. Though difficult to interpret there were trends in favour of the intervention group for physical activity levels as assessed with the IPEQ-W and a reduction in agitated behaviours measured by the ABID (Table 2). The physiological measures (PPA) showed no improvement with the Hill Step Test actually showing a poorer outcome for the intervention group and no change in the control group.

**Table 2:** Comparison of physiological and functional outcomes for person with dementia

**Measure**	**Baseline or 12 wk follow up**	**n**	**Intervention mean ±SD**	**n**	**Control mean ±SD**	**P**^**2**^
**PPA falls risk score**^**1**^	B	11	0.78 (±1.15)	11	1.69(±1.74)	
	F	9	1.42 (±1.63)	11	2.65 (±1.83)	.82
**Near tandem eyes closed**	B	11	5.19 (±3.58)	11	5.71(±3.01)	
	F	9	5.35 (±3.72)	10	6.29 (±3.70)	.32
**Hill step test**	B	11	19.18 (±6.53)	11	14.45(±4.96)	
	F	6	15.0 (±5.12)	8	14.20 (±7.67)	.10
**IPEQ-W (Incidental and planned exercise)**	B	11	20.77 (±11.69)	11	14.41 (±10.57)	
	F	9	32.96 (±18.46)	11	14.53 (±14.91)	.26
**IDDD: (Activities of daily living)**	B	11	46.4 (±8.2)	11	49.4 (±13.8)	
	F	7	49.9 (±11.6)	10	53.7 (±15.9)	.40
**FES-I short form**^**1**^	B	11	10.5 (±4.4)	11	10.0 (±3.0)	
	F	9	8.2 (±1.9)	11	9.4 (±5.4)	.71
**ICON-FES**^**1**^	B	11	51.6 (±21.8)	11	51.3 (±18.9)	
	F	8	47.3 (±18.5)	9	44.6 (±12.8)	.56
**Cornell depression scale**^**1**^	B	11	6.41 (±4.57)	11	5.59 (±5.52)	
	F	6	8.10 (±7.27)	10	6.32 (±4.83)	.29
**Agitated behaviours in Dementia scale**^**1**^	B	11	14.41 (±13.99)	11	14.36 (±16.34)	
	F	7	12.29 (±13.49)	11	14.66 (±15.67)	.58

Notes: *PPA* Physiological profile assessment, *IPEQ-W* Incidental and planned exercise questionnaire – weekly, *IDDD* Interview for deterioration in daily activities in dementia scale, *FES-I* Falls efficacy scale – international, *ICON-FES* Iconographical falls efficacy scale – international.

^1^Lower scores reflect better ability, ^2^Mann–Whitney *U* test using change scores.

The Zarit scale showed a trend in the intervention group for increased burden which was approximately twice that of the control group, though four cases were missing from the intervention group (Table 3). The TMSI was useful in understanding management strategies used by the carers and which ones they took up as a consequence of the intervention. Using routines as a strategy to support function was not endorsed by any carers at baseline: at follow-up 6/7 carers in the intervention group endorsed this strategy; none in the control group. Intervention group carers also reported using briefer instructions with their person with dementia at follow-up (5/7) with none in the control group, and none in either group at baseline. The ICON-FES was preferred by assessors over the FESI because of its visual representation of fear of falling situations.

**Table 3:** Comparison of burden and task management outcomes for carers

**Measure**	**Baseline or follow up**	**n**	**Intervention mean ±SD**	**n**	**Control mean ±SD**	**P**^**2**^
**Zarit carer burden**^**1**^	B	11	15.09 (±9.89)	11	10.45 (±11.79)	
	F	7	19.14 (±12.27)	11	11.64 (±11.48)	.77
**Task strategy management index**	B	11	7.55 (±4.45)	11	8.09 (±4.81)	
	F	7	8.57 (±3.36)	11	6.55 (±4.72)	.64

^1^Lower scores reflect improved outcome, ^2^Mann–Whitney *U* test using change scores.

### Falls

There were fewer falls (n=5) in the intervention than the control group (n=11), (IRR= 0.34 (95% CI 0.06-1.91)). Two people fell in the intervention group and four in the control group (RR = 0.50 (0.11 to 2.19)). Most people in the control group who fell did so in their own home environment with only one fall recorded outdoors in a park. These falls were while participants were conducting everyday activities such as getting dressed (n=3 falls) or transferring/mobilising in the home (n=3 falls) or tripping over obstacles/ steps (n=2 falls). Of the two participants in the intervention group who fell, one fell at home and the other fell while walking a relative’s dog in an unfamiliar environment.

There was one fracture in the control group and none in the intervention group.

## Discussion

The primary aim of the study was to develop and pilot the feasibility of a novel approach to fall prevention for people with dementia. The complexity of the intervention required a determination of the program’s acceptability, feasibility and if it could be achieved from the perspectives of the participants and their carers and the allied health staff who implemented the intervention protocols. Therefore, efficacy or intervention effects cannot be conclusively determined from our results, in keeping with the aims of a feasibility trial [32]. Structural and organisational aspects of the study such as randomisation, blinding, delivery of the intervention, retention of participants and analysis were feasible.

Recruitment for the small number in the pilot was achieved within our time frame and was influenced by the clinical relationships of the investigators. Recruitment challenges included the difficulties of identifying people with dementia in the community and the number of research projects running concurrently in the dementia specific services. In a larger trial, use of a multi-centre approach and recruitment from a wider sample such as health services for older people as well as dementia specific clinical networks would be needed to yield a sufficient sample. Strategies such as advertising through local papers and radio, hospital newsletters and agencies such as Alzheimer’s Associations, and screening at local community centres and GP clinics may successfully recruit larger numbers. All participants in the control group expressed disappointment that they would not be receiving intervention, and the general enthusiasm and positive response to recruitment are perhaps indications of the lack of specific (or individually tailored) community based intervention programs available for people with dementia.

Incomplete follow-up assessments, as here, caused differential reporting and can introduce bias. Our baseline assessments compared to our follow-up assessments were undertaken by research assistants with very different expertise and experience in working with people with dementia. A consideration for future studies might be to ensure all assessors have relevant experience and training in dementia-specific assessment. We also support the importance of further work validating the psychometric properties of the physiological measures for use in this population. Challenges can occur in testing people with dementia using physical measures with issues to do with concentration, test order and the careful use of tactile and visual cueing depending on level of impairment [33,34].

The study sample contained participants with mild dementia but was still a relatively heterogeneous group in terms of both physical and cognitive abilities, as is usual with a community based clinical sample. Despite this they were able to maintain acceptable adherence rates for both interventions, comparable with other large trials [35]. The trend for a reduction in the rate of falls and increased activity in the intervention group despite the small sample size suggest that the intervention shows potential benefits.

Adherence to home safety recommendations was good and consistent with the findings from a large and effective randomised trial [36] which reduced fall rates in a cognitively intact population for those who reported a fall in the previous year. Meta-analysis provides strong evidence [6,8] that home safety interventions targeted to high risk groups are effective in reducing fall risk. In our study, participants made numerous changes which comprised simple recommendations such as securing mats, highlighting step edges, changing footwear, re-arranging furniture to improve clear access and strategies for medication management. There was a trend for higher rates of implementation in those who had a history of falls suggesting, as in previous home safety studies [8], that highest yield for this intervention is likely to come from targeting people with a recent fall. Participants with greater cognitive impairment were also more receptive and evidence suggests that increasing risk of falls is associated with subtle and increasing cognitive impairment [37]. Overall, this intervention component appeared to be acceptable, feasible and of benefit.

Adherence to exercise was also very good and comparable to successful fall trials [35]. However in future studies we would recommend an increase in both frequency and intensity to achieve a clinically meaningful impact on overall risk profile. Heterogeneity of the sample in physical functioning as well as cognitive abilities may have contributed to less intense exercise prescription and progression. Some participants reported no physical activity outside personal care and ADL tasks (for example, minimum IPEQ-W score of 6.8 hrs per week) while another reported exercising extensively (IPEQ-W score of 47 hours per week). This, plus the combination of cognitive status and living status are likely to have impacted on exercise prescription and subsequent upgrading.

A number of recommendations about the exercise protocol can be made. Simple ankle cuff weights have been part of the current successful home based programs with cognitively intact people [35]. Logsdon [12] found that resistance bands were difficult to use with their mild cognitively impaired groups and instead used body weight for resistance. Contrary to this Steinberg et al. [38] used both resistance bands and cuff weights in a home based physical activity program for people with moderate dementia. Consideration needs to be given to how these are introduced and taught. Secondly, a more detailed exercise history obtained at baseline could be incorporated into the exercise prescription to supplement the baseline assessment and used to better tailor the approach to exercise prescription. Finally, a greater choice of alternate exercises which are sufficiently challenging but implemented differently so as to accommodate the different cognitive and social circumstances is recommended. Recently, a supervised group-based intensive exercise program, conducted over three months, has demonstrated the potential to improve strength and functional performance in cognitively impaired older people recruited after rehabilitation and through community nurses [39]. This study did not show a significant reduction in falls, but was not powered to detect fall reductions. Further work is needed to determine whether strength and balance measures can be improved in stable community populations and whether these improvements translate into reductions in falls and fall-injury.

We recommend an integrated therapy approach with refined protocols where occupational therapist and physiotherapist are trained to implement both components. Such team support would enhance the delivery of the intervention with carers and ensure adequate progression of participants towards intervention goals. This may also assist in reducing the number of visits required. This team approach is plausible and its application has been demonstrated in a restorative functional independence project [40].

The tailored approach accommodating both physical and cognitive abilities was a key component of this intervention, enabling people with a wide range of abilities to fully participate. The use of the Allen’s model was useful in guiding the modification of cognitive demands of the program. The simple formats of the separate booklets for exercises and for home safety were enthusiastically received by participants and carers. Our modifications included simple statements reminding participants what each exercise was targeting and improved and simplified methods for recording adherence.

Personal goals achieved were not always directly related to falls prevention. For example, one participant started walking to the bus on her own as she felt more confident; another participant who was initially very resistant to allowing community services into her home and had a long history of rejecting help was able to allow limited community care staff into her home in addition to the intervention staff; and a number of participants commenced other community services such as meal provision. These outcomes might be better represented in a participation measure or with qualitative methodology. Other needs such as the lack of activities for people with dementia that are appropriate, interesting and accessible and not dependent on carer for transport were identified by all dyads.

Within our sample multiple personal issues and circumstances arose yet participants were very committed and engaged in the program with few cancellations. This feasibility study is the first falls study to include people with dementia in a home based intervention. Given the practical challenges for carers and people with mild dementia to attend centre based programs such alternatives need to be developed and rigorously tested.

The trend towards increased burden for carers of the intervention group may have been incidental or a direct result of their engagement in the intervention. It was our observation that many of the carer stressors were related to other life roles, for example, family and work issues. It may be that both participants and carers are ‘reminded’ of the dementia and its consequences, for example, discussion of home safety recommendations may have had implications for decision making about future care needs, which is also stressful. We noted that some carers could benefit from additional education regarding management and communication skills. An environmental skills building approach that includes training of the carers in management and coping skills have been shown to reduce caregiver burden [41]. Further, Teri et al. [13] successfully combined carer training in behavioural management techniques with home based exercise to reduce functional dependence and delay institutionalisation. This indicates there are opportunities to enhance this intervention.

There are a number of limitations to this study. The sample of participants lived in the community and had mild dementia and thus the intervention protocol has not been tested in people with a moderate-severe dementia. While the two groups were not significantly different at baseline there were missing data which is likely to affect the direction of results. The IPEQ-W has not been validated for people with cognitive impairment and thus we relied on carer report where possible. While fall reporting processes are the same for control and intervention, it may be underestimated if the person with dementia does not remember to report all falls, though as this was a group with mild dementia this would have been less of an issue. There were more control participants who lived alone which could have led to differential reporting bias as carers assisted with completing fall calendars. The sample recruited included people who did not have a history of a fall in the previous year and this may have influenced motivation to adhere to the intervention.

While both intervention components were acceptable both offer challenges with this group of people in terms of comprehensiveness and intensity. We believe that for this high-risk population a multi-component intervention that can be tailored to cognitive and functional capacity and be relevant to carer and participant life situation is required.

## Conclusions

The pilot demonstrated that a home-based tailored intervention was feasible and acceptable to people with mild dementia and their carers. Future research on fall prevention for people with dementia should consider the following: recruitment for a larger trial will require multiple approaches; assessors should have dementia-specific experience in interviewing to maximise ease and reliability and completeness of data collection; the intensity in intervention components be balanced by integrating therapy approaches and by progressing the balance and strength opportunities; a fall intervention needs to consider the carers’ needs and provide specific carer training session in behaviour management and communication skills as these concerns dominate daily care. We recommend that a refined and enhanced intervention be tested in a randomised trial powered to detect an effect on falls as an outcome measure.

## Competing interests

The authors declared that they have no competing interests.

## Authors’ contributions

JW made substantial contributions to the development of the intervention, conducted baseline assessments and the occupational therapy aspects of the intervention, conducted the data entry and data analysis and drafted the manuscript. LC made substantial contributions to the conception and design of the study, contributed to data analysis and interpretation and was involved in manuscript drafting. JC made substantial contribution to the conception and design of the study, interpretation of data and contributed to manuscript revision. MT conducted the physiotherapy intervention and contributed to the revision of the manuscript. HB, SL & LG contributed to the study design and critical revisions of manuscript. All authors read and approved the final manuscript.

## Pre-publication history

The pre-publication history for this paper can be accessed here:

http://www.biomedcentral.com/1471-2318/13/89/prepub

## BMC geriatrics

http://www.biomedcentral.com/bmcgeriatr/about/
